# The Brazilian Version of the Edmonton Symptom Assessment System (ESAS) Is a Feasible, Valid and Reliable Instrument for the Measurement of Symptoms in Advanced Cancer Patients

**DOI:** 10.1371/journal.pone.0132073

**Published:** 2015-07-08

**Authors:** Carlos Eduardo Paiva, Luciana Lopes Manfredini, Bianca Sakamoto Ribeiro Paiva, David Hui, Eduardo Bruera

**Affiliations:** 1 Department of Clinical Oncology, Barretos Cancer Hospital, Barretos, São Paulo, Brazil; 2 Learning and Research Institute, Barretos Cancer Hospital, Barretos, São Paulo, Brazil; 3 Cancer Hospital Children and Youth President Luiz Inacio Lula da Silva, Barretos Cancer Hospital, Barretos, São Paulo, Brazil; 4 Departments of Palliative Care and Rehabilitation Medicine, The University of Texas M. D. Anderson Cancer Center, Houston, Texas, United States of America; Iranian Institute for Health Sciences Research, ACECR, ISLAMIC REPUBLIC OF IRAN

## Abstract

**Purposes:**

To develop and validate a Portuguese version of the Edmonton Symptom Assessment System (ESAS) in Brazilian patients with advanced cancer.

**Methods:**

The ESAS was translated and then back translated into Portuguese in accordance with international guidelines. The final version was approved by an Expert Committee after pilot testing on 24 advanced cancer patients. Subsequently, we evaluated the time to complete the assessment, the number of unanswered items, internal consistency, convergent validity, and known-group validity in a sample of 249 advanced cancer patients who completed the ESAS along with the European Organization for Research and Treatment of Cancer Core Quality of Life Questionnaire (EORTC QLQ-C30), Hospital Anxiety and Depression Scale (HADS), and Epworth Sleepiness Scale (ESS). A total of 90 clinically stable patients were retested after 4 to 96 hours (test-retest reliability), and 80 patients answered the ESAS after 21 (±7) days to measure scale responsiveness using an anchor-based method.

**Results:**

The ESAS was completed in a mean time of only 2.2 minutes. The internal consistency was good (Cronbach's alpha = 0.861), and the removal of single items did not change the overall alpha value. For convergent validity, Spearman’s correlation coefficients between the ESAS symptom scores and the corresponding EORTC QLQ-C30 and ESS symptom scores ranged between 0.520 (95% CI = 0.424–0.605) and 0.814 (95% CI = 0.760–0.856), indicating moderate to strong correlations. Test-retest reliability values were considered adequate, with intraclass correlation coefficients ranging from 0.758 (95% CI = 0.627–0.843) to 0.939 (95% CI = 0.905–0.960).

**Conclusions:**

ESAS is a feasible, valid and reliable multi-symptom assessment instrument for use in Brazil.

## Introduction

Patients with advanced cancer frequently report multiple concomitant symptoms [1,2] that negatively affect their quality of life [3]. Therefore, the adequate control of symptoms is an essential part of supportive care in oncology. The number of symptoms found using systematic assessments has been shown to be tenfold higher than the number that are voluntarily reported [2]. Thus, routine and systematic symptom assessments with a validated symptom assessment tool should occur during patient visits to oncology clinics [4,5].

A number of symptom screening instruments have been developed, including those that target single and multiple symptoms [6,7]. The Edmonton Symptom Assessment System (ESAS) is a widely used, multiple-symptom assessment instrument that was first developed in 1991 by Bruera et al. to audit the symptoms of patients receiving palliative care [8]. Originally, the ESAS investigated eight symptoms using visual analog scales (0–100 mm). However, these scales have undergone several changes since they were first implemented. The most recent version of the ESAS evaluates 10 common symptoms using categorical numbered scales (0–10) [9,10]. The ESAS has been translated and validated in several languages and cultures [8–16]. Although it is frequently adapted in medical practice and research in Brazil, its psychometric properties have not been formally investigated to date.

Thus, we developed a Brazilian version of the ESAS by performing a translation and cultural adaptation and subsequently tested it on a sample of advanced cancer patients to measure its psychometric properties.

## Methods

### Ethics Statement

This study complied with the ethical standards of the Declaration of Helsinki and Brazilian National Health Council resolution no. 466/2012. It was approved by the Research Ethics Committee of the Barretos Cancer Hospital (BCH; Barretos, SP, Brazil) under number 100.425. Each of the included patients voluntarily signed a consent-to-participate form.

### Study design and selection of participants

The present cross-sectional study employed methods for the translation and validation of assessment instruments. To be eligible, patients had to have been diagnosed with advanced cancer (locally advanced, relapsed or refractory or metastatic disease), at least 18 years old, and a native Portuguese-speaking Brazilian. Patients with any confusion, uncontrolled psychiatric disease, cognitive dysfunction, or any other disabling disease that could hinder their ability to answer the study questionnaires were not eligible to participate.

### Phase I—Translation process

The ESAS is a well known symptom-intensity tool for assessing ten common symptoms (pain, fatigue, nausea, anxiety, depression, drowsiness, anorexia, well-being, dyspnea, and sleep disturbance) in cancer care [8]. The severity of each symptom is rated from 0 to 10, with 0 indicating that the symptom is absent and 10 describing the worst possible severity [17]. The total symptom distress score (TSDS) represents the sum of all ESAS symptoms (from 0 to 100) [8,18].

Five different versions of the ESAS (V1A, V1B, V1C, V1D, and V1E) were obtained by translating them from their original English versions into the target Portuguese/Brazil language. Translations were performed by five different Brazilian native speakers who were also fluent in English. These included a radiation oncologist, a medical oncologist, two engineers, and an English teacher; both oncologists were familiar with the scale, but had never had used it. The translation panel for the reconciliation of these five versions consisted of four authors (BSRP, LLM, EMB, and BSRP), who analyzed the detailed translations and synthesized them into a grouped version (V1A-E). Two native North American professional translators, who spoke Portuguese/Brazil fluently but had no prior knowledge of the ESAS, subsequently performed a back translation from the V1A-E version into English. Again, the same translation panel for reconciliation synthesized the two translations into a grouped version (V2A-B). A bilingual Expert Committee that was composed of five members (a medical oncologist, registered nurse, dentist, occupational therapist, and psychologist), all of whom had expertise in instrument validation studies, was assembled. Out of this group, the physician, nurse and occupational therapist had extensive experience in palliative care practice and research. This committee analyzed all of the translation documents using a structured protocol that has been described elsewhere [19] and produced the final adapted version of the ESAS to be pre-tested. Cognitive debriefing interviews on the Brazilian version of ESAS were conducted with 24 advanced cancer patients of different educational backgrounds who were representative of the target population. These patients were questioned about confusion, embarrassment, and comprehension difficulties, and when necessary, they were given suggestions. Patient understanding of the ESAS was also graded according to the interviewer’s opinion.

### Phase II–Psychometric evaluation

#### Informed consent process and data collection

A research coordinator from the Research Support Center (Barretos, SP, Brazil) identified potential participants via convenience sampling at the Clinical Oncology Department (outpatient clinics and inpatient ward) of the BCH by checking eligibility criteria from the medical charts. In sequence, interviewers contacted potential participants in person and informed them about the objectives and procedures of the study. Then, after asking simple initial questions (“what is your name?”, “where are you from?”, “what kind of disease are you treating?” and “which treatment are you receiving?”), the interviewers confirmed each patient’s ability to communicate in Brazilian Portuguese and identified cases of evident cognitive dysfunction. Those who voluntarily agreed to participate in the study completed the evaluation instruments.

All of the patients who were included in the validation phase of this study completed the ESAS, Hospital Anxiety and Depression Scale (HADS), Epworth Sleepiness Scale (ESS) and European Organization for Research and Treatment of Cancer Core Quality of Life Questionnaire (EORTC QLQ-C30). These instruments could be completed in either a self-administered or interviewer-assisted manner, depending on the choice of the participant. To assess test-retest reliability, 84 clinically stable participants were subjected to a second interview 4 to 96 hours later. To be considered clinically stable, a patient’s Karnofsky performance status at both of the two visits needed to be within 10% of each other. Following this, to assess the responsiveness (the ability of the instrument to detect changes in symptoms) of the ESAS, 80 patients retook the test after 21 (±7) days. For each symptom evaluated by the ESAS, participants answered as to whether it was better than, the same as, or worse than it was during the first evaluation. Data were collected from August 2012 to March 2014.

#### Validation instruments

*EORTC QLQ-C30*. The 30-item EORTC QLQ-C30 (version 3.0) includes a scale that measures global health, five functioning scales (physical, role, emotional, cognitive, and social), three symptom scales (fatigue, nausea/vomiting, and pain), six single items addressing common symptoms (dyspnea, sleep disturbance, appetite loss, constipation, and diarrhea), and an additional item measuring financial difficulties. Items are rated on a Likert scale from 1 (not at all) to 4 (very much), with the exception of two global health items, which are rated from 1 (very poor) to 7 (excellent). All of the items are linearly transformed to a 0–100 scale. For the functioning and global quality of life scales, a higher score represents a better quality of life. In contrast, a higher score on the symptom scale represents greater symptom severity [20]. The EORTC QLQ-C30 has been previously validated in Brazil [21]. In the present study, Cronbach’s alpha ranged from 0.622 (cognitive functioning) to 0.852 (global health).

*Hospital Anxiety and Depression Scale*. The HADS questionnaire consists of 14 items with a 4-point Likert-type scale ranging from 0 (minimally present) to 3 (maximally present). It is commonly used to assess anxiety and depression among individuals with cancer and has been validated in Brazil [22,23]. HADS-A and HADS-D scores range from 0 to 21, with higher scores indicating greater distress. In the present study, Cronbach’s alphas were 0.78 (HADS-A) and 0.82 (HADS-D).

*Epworth Sleepiness Scale*. The ESS was developed to identify the occurrence of excessive daytime sleepiness. The ESS consists of 8 items that are rated using a 4-point scale (0–3) [24]. The higher the score, the greater the daytime sleepiness. The ESS has been previously validated in Brazil [25]. In the present study, Cronbach’s alpha was 0.808.

*Karnofsky Performance Status*. The KPS is an 11-point rating scale that ranges from 0 (dead) to 100 (normal function) and was initially developed to assess functional status in patients with cancer [26]. It has been used in previous Brazilian studies [27,28].

### Statistical analysis

Clinical utility was estimated by measuring the mean (standard deviation [SD]) time to complete the ESAS and the number of missing items per ESAS item. Internal consistency was assessed using Cronbach’s alpha coefficient, and a value between 0.70 and 0.95 was considered adequate [29]. Test-retest reliability was measured using the intraclass correlation coefficient (ICC) at 4 to 96 hours after the first evaluation; a value ≥0.70 was considered adequate [29]. Regarding convergent validity, we hypothesized that the results of the HADS, EORTC QLQ-C30, and ESS, all of which measure similar symptoms as the ESAS, would be at least moderately correlated with the results of the ESAS. Pearson correlation coefficients of >0.6, 0.4–0.6, and <0.4 were considered strong, moderate, and poor correlations, respectively [30]. The known-group validity analysis was performed to evaluate the extent to which the instrument was able to discriminate between clinical subgroups of patients. The ESAS symptom scores were compared between groups of patients with different KPS values (≤70% versus >70%) and treatment locations (inpatient versus outpatient). The non-parametric Mann-Whitney test was used for these comparisons. Responsiveness analyses were carried out using an anchor-based strategy. After 21 (±7) days, patients were asked to classify their symptoms as worse than, the same as, or better than those experienced during their first study visit. Following this, median values were calculated for each category (worse, the same and better) and for each specific ESAS symptom. Differences were evaluated using a non-parametric, two-sided Wilcoxon signed rank test.

All statistical analyses were performed using SPSS software version 20.0 (Chicago, IL, USA). P-values of less than 0.05 were considered statistically significant.

## Results

### Phase I–Translation process

**S1 Table** describes the original version, the translated version, the back-translated version, the final adapted version, and core commentaries from the Expert Committee. During the translation process, the committee questioned the best terms in Portuguese to describe the concepts of fatigue and depression. Thus, we conducted another study to search for the best terms [31]. The findings from this study were then sent to the members of the Expert Committee; each one had the autonomy to decide whether or not to use the results of the aforementioned study. The ESAS was well comprehended by all of the patients. According to the interviewers’ perceptions, 1 (1/24, 4.1%), 2 (2/24, 8.3%), and 3 (3/24, 12.5%) patients only partially understood the fatigue, nausea and drowsiness items, respectively. However, no modification was necessary after the pre-testing, and the panel decided to use the final version of the ESAS (**S2 Table**) in the psychometric validation study.

### Phase II–Psychometric evaluation

Originally, 293 patients were invited to participate in the study. Of them, 1 was considered a screening failure (patient did not presented advanced disease), and 43 refused to participate. Therefore, the final sample population comprised 249 advanced cancer patients.

The mean (SD) age was 55.1 (12.6) years. The mean (SD) KPS value was 78.1 (13.2). The majority of the patients were women (158/249, 63.5%), most of who had less than 8 years of formal education and actively worked (174/249, 70.0%). The mean (SD) wage was 2.94 (3.28) Brazilian minimum wages. The most common primary cancer types were breast (n = 85, 34.1%) and colorectal (44/249, 17.7%). There were 218 (218/249, 87.6%) and 50 (50/249, 20.1%) patients with distant metastasis and locally unresectable recurrence, respectively. The majority of the patients were receiving palliative chemotherapy (208/249, 83.5%). The demographic and clinical characteristics of the patients are detailed in **Table 1**.

**Table 1:** Demographic and clinical characteristics of the patients (n = 249).

Characteristic	N (%)
*Age*, *mean (SD)*	55.1 (12.6)
*Gender*	
Male	158 (63.5)
Female	91 (36.5)
*Years of formal education*	
None	21 (8.4)
1–8	124 (49.8)
8–11	65 (26.1)
>11	39 (15.7)
*Work activity*	
Active	174 (69.9)
Inactive	75 (30.1)
*Financial income* ^a^, mean (SD)	2.93 (3.28)
*Primary tumor site*	
Cervix	14 (5.6)
Colorectum	44 (17.7)
Endometrium	8 (3.2)
Esophagus	9 (3.6)
Stomach	17 (6.8)
Breast	85 (34.2)
Ovary	8 (3.2)
Pancreas	7 (2.8)
Prostate	22 (8.8)
Lung	9 (3.6)
Others^b^	26 (10.4)
*Distant metastasis*	
Yes	218 (87.6)
No	31 (12.4)
Locoregional unresectable recurrence	
Yes	50 (20.1)
No	199 (79.9)
*Metastasis site*	
Liver	56 (22.5)
Bone	63 (25.3)
Peritoneum	25 (10.0)
Pleural/lung	21 (8.4)
Other	21 (8.4)
*KPS*, *mean (SD)*	78.1 (13.2)
*Current treatment*	
Palliative chemotherapy	208 (83.5)
Palliative radiotherapy	2 (0.8)
Palliative care only	39 (15.7)

^a^Brazilian minimum wages (R$).

^b^Unknown primary (n = 6), skin (n = 3), penis (n = 1), sarcoma (n = 5), testis (n = 2), uterine (n = 1), gallbladder (n = 3), vulvar (n = 1), and head and neck (n = 4).

The lowest prevalence and mean symptom scores were nausea (23.3% and 0.99, respectively), dyspnea (29.3% and 1.39, respectively) and depression (26.5% and 1.26, respectively). The highest prevalence and mean symptom scores were sleep disturbance (61% and 3.2, respectively), anxiety (65.5% and 3.15, respectively) and drowsiness (60.5% and 3, respectively) (**Table 2**).

**Table 2:** Mean and median scores, missing values, and percentages of maximum and minimum scores.

Symptom ESAS	Symptom prevalence	Mean (SD)	Cronbach’s α, if item was excluded	ICC (95% CI)
Pain	127 (51.0)	2.64 (3.25)	0.85 (0.82–0.88)	0.89 (0.83–0.93)
Fatigue	143 (57.4)	2.82 (3.03)	0.84 (0.80–0.87)	0.78 (0.66–0.86)
Nausea	58 (23.3)	0.99 (2.11)	0.87 (0.84–0.89)	0.87 (0.80–0.92)
Depression	66 (26.5)	1.26 (2.49)	0.85 (0.82–0.88)	0.89 (0.84–0.93)
Anxiety	162 (65.5)	3.15 (3.20)	0.85 (0.82–0.87)	0.86 (0.79–0.91)
Drowsiness	151 (60.5)	3.00 (3.16)	0.85 (0.81–0.87)	0.76 (0.63–0.84)
Loss of appetite	123 (49.4)	2.47 (3.1)	0.85 (0.82–0.87)	0.83 (0.74–0.89)
Feeling of well-being	138 (55.4)	2.58 (2.84)	0.83 (0.80–0.86)	0.83 (0.74–0.89)
Dyspnea	73 (29.3)	1.39 (2.54)	0.85 (0.82–0.87)	0.84 (0.75–0.89)
Sleep disturbance	152 (61.0)	3.20 (3.23)	0.85 (0.83–0.88)	0.80 (0.70–0.87)
TSDS	NA	23.45 (19.43)	NA	0.94 (0.90–0.96)

Abbreviations: TSDS = total symptom distress score; and NA = not applicable.

#### Instrument’s feasibility

The ESAS was completed in a mean (SD) time of 2.24 (1.36) minutes. Out of 249 questionnaires administered, only 1 non-response item was observed, for which the patient did not understand the meaning of the word drowsiness (“sonolência”).

#### Internal consistency

Cronbach’s alpha coefficient was 0.86 (95% confidence interval [CI] = 0.83–0.88). No single item significantly modified the internal consistency of the scale when deleted (**Table 2**).

#### Test-retest reliability

The general test-retest ICC value was 0.94 (0.90–0.96). When considering isolated items, the lowest values were observed for drowsiness (0.76, 95% CI = 0.63–0.84) and fatigue (0.78, 95% CI = 0.66–0.86) (**Table 2**).

#### Convergent validity

Convergent validity was assessed by determining the correlation between each ESAS symptom and a specific related measure from another validated instrument. All previously hypothesized correlations were further confirmed. In general, we observed strong correlations (r>0.6), with the exception of the following moderate correlations: ESAS-depression and emotional functioning (r = -0.57; 95% CI = -0.49−0.64), ESAS-depression and HADS-D (r = 0.52; 95% CI = 0.42–60), and ESAS-drowsiness and ESS global score (r = 0.55; 95% CI = 0.44–0.64) (**Table 3**).

**Table 3:** Convergent analyses (n = 249).

ESAS Symptoms	Instrument	Item	Correlation coefficient (95% CI)^a^
Pain	EORTC QLQ-C30	Pain	0.75 (0.67; 0.80)
Fatigue	EORTC QLQ-C30	Fatigue	0.75 (0.68; 0.80)
Nausea	EORTC QLQ-C30	Nausea and vomiting	0.66 (0.55; 0.75)
Depression	HADS-D	Depression	0.52 (0.42; 0.60)
	EORTC QLQ-C30	Emotional functioning	-0.57 (-0.49; -0.64)
Anxiety	HADS-A	Anxiety	0.65 (0.57–0.72)
	EORTC QLQ-C30	Emotional functioning	-0.62 (-0.52; 0.70)
Drowsiness	Epworth Sleepiness Scale	Global score	0.55 (0.44; 0.64)
Loss of appetite	EORTC QLQ-C30	Loss of appetite	0.70 (0.62; 0.77)
Well-being	EORTC QLQ-C30	Global health	-0.61 (-0.52; -0.70)
Dyspnea	EORTC QLQ-C30	Dyspnea	0.79 (0.70; 0.87)
Sleep disturbance	EORTC QLQ-C30	Sleep disturbance	0.63 (0.54; 0.70)
TSDS	EORTC QLQ-C30	Global health	-0.63 (-0.54; -0.71)
	EORTC QLQ-C30	Global functioning	-0.74 (-0.68; -0.80)
	EORTC QLQ-C30	Global symptoms	0.81 (0.76; 0.86)

TSDS = total symptom distress score.

^a^All p-values are <0.001.

#### Known-group validity

As expected, the inpatients reported higher median symptom scores than the outpatients for all individual ESAS symptoms. Regarding the TSDS, the median values of the outpatients and inpatients were 16 and 37, respectively (**Table 4**). The same pattern was observed when comparing the median scores of the ESAS symptoms and the ESAS-TSDSs of the patients based on their KPS; all analyses revealed that the patients with low performance statuses (KPS≤70%) had higher scores than those with better performance statuses (KPS>70%) (**Table 4**).

**Table 4:** Known-group validation analyses (n = 249).

ESAS symptoms	Place of treatment		p-value^1^	KPS		p-value^1^
	Median (p25-p75)			Median (p25-p75)		
	Outpatient (n = 200)	Inpatient (n = 49)		≤70% (n = 99)	>70% (n = 148)	
Pain	0 (0–4.75)	5 (0–9)	<0.001	5 (1–8)	0 (0–2)	<0.001
Fatigue	1.5 (0–5)	5 (0–6.5)	0.018	5 (1–7)	0 (0–3)	<0.001
Nausea	0 (0–0)	0 (0–5)	0.005	0 (0–3)	0 (0–0)	0.001
Depression	0 (0–0)	1 (0–6)	<0.001	0 (0–5)	0 (0–0)	<0.001
Anxiety	2 (0–5)	5 (1–5)	0.005	5 (1–6)	1 (0–5)	<0.001
Drowsiness	2 (0–5)	5 (3–7)	<0.001	5 (3–7)	0 (0–3)	<0.001
Loss of appetite	0 (0–4)	5 (0–5)	<0.001	3 (0–5)	0 (0–3)	<0.001
Well-being	1 (0–5)	4 (0–5)	0.001	5 (0–6)	0 (0–3)	<0.001
Dyspnea	0 (0–1)	0 (0–5.5)	0.011	0 (0–5)	0 (0–0)	<0.001
Sleep disturbance	2 (0–5)	5 (2–5)	0.007	5 (0–6)	1 (0–5)	0.001
TSDS	16 (6–31)	37 (17–53.5)	<0.001	37 (16–53)	12 (5–23)	<0.001

^1^Mann-Whitney test.

Legend: TSDS = total symptom distress score.

#### Responsiveness

It was expected that the patients with no perception of change from their first to their second study visit would have no statistically significant changes in their scores. This was confirmed for all ESAS symptoms, with the exception of dyspnea, for which the score for the first visit was slightly higher than that for the second (p = 0.029). Patients who reported feeling better at the second visit had significantly lower ESAS scores for pain (p = 0.038), fatigue (p = 0.008), depression (p = 0.027), loss of appetite (p = 0.001), feeling of well-being (p = 0.037), and sleep disturbance (p = 0.024) than at the first visit. The only ESAS symptom score that was significantly altered in the patients who perceived symptom deterioration was that for pain (p = 0.042). However, in general, a low number of patients were classified as having symptom deterioration, which could have impacted the statistical analyses (**Table 5**).

**Table 5:** ESAS responsiveness analyses (n = 90).

ESAS symptoms	Health status at follow-up visit^1^	N	Median (p25-p75)		p-value^2^
			First consultation	Follow-up consultation	
Pain	Better	26	1 (0–5)	0 (0–1)	**0.038**
	The same	49	0 (0–4)	0 (0–2)	0.403
	Worse	7	0 (0–5)	5 (5–8)	**0.042**
Fatigue	Better	28	2 (0–4)	0 (0–2.75)	**0.008**
	The same	43	0 (0–5)	0 (0–5)	0.795
	Worse	11	5 (2–6)	5 (5–7)	0.171
Nausea	Better	24	0 (0–0.75)	0 (0–0)	0.121
	The same	53	0 (0–0)	0 (0–0)	0.969
	Worse	5	0 (0–3.5)	5 (2.5–7.5)	0.102
Depression	Better	21	0 (0–3)	0 (0–0)	**0.027**
	The same	54	0 (0–0)	0 (0–0)	0.239
	Worse	7	2 (0–5)	5 (0–6)	0.109
Anxiety	Better	19	1 (0–6)	2 (0–4)	0.494
	The same	58	2 (0–5)	0.5 (0–4)	0.233
	Worse	3	5 (0-)	8 (5-)	0.317
Drowsiness	Better	20	2 (0–6)	1.5 (0–4.5)	0.397
	The same	54	1.5 (0–3)	1 (0–4.25)	0.873
	Worse	6	3 (1.5–5)	7 (2.25–8.5)	0.104
Loss of appetite	Better	29	1 (0–5.5)	0 (0–1)	**0.001**
	The same	40	0 (0–3)	0 (0–1)	0.341
	Worse	12	6 (2–9.25)	4.5 (3–7.5)	0.573
Well-being	Better	27	1 (0–6)	0 (0–4)	**0.037**
	The same	48	0 (0–2.75)	0 (0–2)	0.708
	Worse	6	5 (3.75–5.75)	5 (3.5–7)	0.715
Dyspnea	Better	15	0 (0–2)	0 (0–0)	0.173
	The same	63	0 (0–1)	0 (0–0)	**0.029**
	Worse	3	0 (0-)	7 (5-)	0.109
Sleep disturbance	Better	27	3 (0–7)	0 (0–3)	**0.024**
	The same	49	2 (0–5)	1 (0–3.5)	0.266
	Worse	5	5 (4–7)	5 (3.5–8.5)	0.581

Significant results are marked in bold letters.

## Discussion

The majority of patients with advanced cancer are exclusively treated by medical oncologists during most of the time that they receive palliative chemotherapy. Although the early integration of palliative care in the treatment of advanced cancer patients is recommended, it is typically only offered late during the course of the disease, even at comprehensive cancer centers [32]. A key approach to improving the health-related quality of life of cancer patients undergoing chemotherapy is the timely and effective control of uncomfortable symptoms [18,33]. Patients with advanced cancer frequently report many symptoms concomitantly; thus, the proper screening of these symptoms is essential in oncology care [5]. In this study, the ESAS was completed by advanced cancer patients, most of whom were undergoing palliative chemotherapy. In Brazil, the ESAS has been widely used in clinical practice and research, even without proper psychometric validation. However, it is rarely used in routine medical oncology. In our opinion, appropriate strategies for the screening of symptoms and the implementation of institutional flowcharts for the management of these symptoms should be a priority for excellence in clinical oncology services. The ESAS may play a valuable role in this endeavor. In our study, the ESAS was easily completed in a mean time of only 2.2 minutes.

Based on a previous "think-aloud" study of 20 advanced cancer patients, the ESAS was revised (ESAS-r) by Canadian researchers [34]. The revised version is easier to understand than the original as determined by Watanabe et al. [35]. Our results contradict the need to modify the ESAS, showing that it was easy to understand, and possessed only one unanswered item (due to lack of comprehension). Interestingly, our Brazilian version of ESAS contains two added words, which were inserted in brackets, for the definitions of fatigue and nausea. We believe that the addition of these words in brackets facilitated the understanding of these items. The ESAS-r was translated simultaneously and independently by another Brazilian research group [36]. Interestingly, the final versions of both instruments are quite similar, which validates the translation and adaptation processes performed by both studies. However, the psychometric properties of the Brazilian version of the ESAS-r have not yet been evaluated.

The translation process was conducted according to guidelines proposed by Guillemin et al. [37]. However, we conducted another study concurrent with the translation process to define the best terms in Portuguese (Brazil) to explain the concepts of "depression" and "fatigue" [31], as was performed for the Spanish validation of the ESAS [10]. It was difficult to create a conciliatory version (T1AB) with regard to these terms because several words were initially considered equally appropriate. Generally, the members of the Expert Committee decided on the best terms or phrases to be used in the translated questionnaire based on their personal opinions. However, the results of our simultaneous study were made available to the members of the Expert Committee, who had the autonomy to decide whether to use the results during the process of translating and adapting the ESAS. This method of empirically defining terms could be more frequently used in validation studies, depending on the unresolved issues encountered during translation processes. This method is feasible and appears to facilitate expert decisions.

The reliability of the ESAS was evaluated by calculating the internal consistency and the test-retest values. The internal consistency values that were calculated were considered adequate and were in accordance with those reported by previous ESAS validation studies [9,14,15,38]. Regarding test-retest reliability, our results were also considered quite adequate. We expected ICC values to be above 0.7, which were observed for all ESAS items and TSDSs. Although the ICC 95% CIs for fatigue, drowsiness and sleep disturbance included the lower limit of significance that we adopted, their values were all very close to 0.7.

The validity of the ESAS was assessed using convergent validity and also by comparing symptom scores between known distinct groups of patients. With regard to convergent validity, the ESAS symptom scores were correlated with items or subscales from other questionnaires that measured the same constructs and were previously validated in Brazil. In general, we observed significant correlations, and most were higher than expected (r>0.6). Appropriate correlations of ESAS scores with other instruments have been demonstrated in previous validation studies [9,11,16]. Known-group validation is another important psychometric property that is commonly evaluated in validation studies. Instruments with adequate sensitivities are required to detect clinical differences between groups that are known to be different [30]. Our results confirmed the previous hypothesis that median ESAS values are significantly higher in inpatients and those with worse functional statuses compared to outpatients and those with better functional statuses. Consistent with our findings, previous studies [10,16,38] have shown that the ESAS is a tool with adequate sensitivity for identifying patients with different conditions.

Responsiveness is the ability to detect clinically important differences that occur over time [30]. In the present study, we re-evaluated the ESAS scores after 14–28 days. The scores of patients in three different groups were analyzed as a function of their perceived changes in clinical conditions, which were classified as better, worse, or the same. In general, the median scores of the patients who considered themselves better were improved, and those of the patients who reported a worsened condition were decreased. However, we observed statistically significant differences in only some of the analyses, which can be explained by the small sample size. The patients who considered themselves better (of whom there was a greater number and therefore a larger sample size) had statistically significantly lower scores for pain, fatigue, depression, lack of appetite, feeling of well-being and sleep disturbance. The difference in pain was so profound in those who considered themselves worse that even with a sample size of only seven patients it was possible to obtain a statistically significant difference. From a statistical point of view, the inclusion of a higher number of patients with worse clinical conditions (reporting greater symptom burdens) would be beneficial. In these patients, symptomatic worsening is more frequent, and improvement, when it occurs, is supposedly more evident. Previous studies assessing the ESAS have had difficulties in demonstrating the responsiveness of the scale statistically [9,10,16]. A strength of our study is the evaluation of responsiveness using an anchor-based method. To the best of our knowledge, ours is the first validation study of the ESAS using this methodology in the analysis of responsiveness.

The present study has several limitations. One limitation is that we included patients with better functional conditions who were mostly in outpatient chemotherapy. Thus, we might have overestimated the instrument’s feasibility because patients in worse functional condition might experience greater difficulties in understanding or completing the ESAS items. However, even in considering these aspects, our results regarding its feasibility justify its routine use for the screening of symptoms in the oncology setting. Another limitation of this study is the small sample size that was used in the analysis of responsiveness. Because most patients showed clinical improvement or stability, our analysis was limited by the small proportion of patients who reported a worsening of symptoms. Our center is currently involved in a separate large, multicenter study to evaluate the responsiveness of the ESAS and to determine the minimal clinically important difference values.

## Conclusions

The ESAS should be considered a reliable and valid instrument for use in Brazil to assess symptoms in advanced cancer patients. Our results revealed that this tool is easy to understand and can be quickly completed, suggesting that it could be used in routine practice in palliative care as well as in medical oncology clinics. A larger, ongoing study is being performed to confirm our findings regarding the responsiveness of the ESAS.

## Supporting Information

S1 Table Summary of the original, forward-translated, back-translated, and final adapted versions and core comments from the Expert Committee. (DOC)

S2 Table Final version of the Edmonton Symptom Assessment System (ESAS) in Portuguese from Brazil. (DOC)
