# Supplementary material for: The Brazilian Version of the Edmonton Symptom Assessment System (ESAS) Is a Feasible, Valid and Reliable Instrument for the Measurement of Symptoms in Advanced Cancer Patients
Source: PLoS One. 2015 Jul 8;10(7):e0132073. doi: 10.1371/journal.pone.0132073 (PMC4496067; doi:10.1371/journal.pone.0132073)
Supplement: S1 Table — (DOC) [file pone.0132073.s001.doc]

**S1 Table.** Summary of the original, translated, and final adapted versions and core comments from the Expert Committee.

| **Original version**  **(English)** | **Forward translated version**  **(Portuguese)** | **Back-translation version (English)** | **Expert Committee suggestions** | **Final adapted version**  **(Portuguese)** |
| --- | --- | --- | --- | --- |
| Edmonton Symptom Assessment Scale | Escala de Avaliação de Sintomas de Edmonton | Edmonton Symptom Assessment Scale | Include the acronym of the scale after the official name. | Escala de Avaliação de Sintomas de Edmonton (ESAS) |
| Please circle the number that best describes your symptoms in the last 24 hours: | Por favor, circule o número que melhor descreve os seus sintomas nas últimas 24 horas: | Please circle the number that best describes your symptoms in the last 24 hours: | The need for a brief explanation of the purpose of the instrument was argued. | Esta é uma escala de avaliação de sintomas. Você responderá a 10 itens com respostas que variam de 0 (mínima intensidade) a 10 (máxima intensidade). Por favor, circule o número que melhor descreve os seus sintomas nas últimas 24 horas: |
| No pain/Worst pain imaginable | Sem dor/Pior dor possível | No pain/Worst pain possible | The translated version was maintained (no changes). | Sem dor/Pior dor possível |
| No fatigue/Worst fatigue imaginable | Sem fadiga/Pior fadiga possível^a^ | No fatigue/Worst fatigue possible | Considering both terms (tiredness and weakness) as acceptable, the term weakness was added in parentheses after the term fatigue to facilitate patient comprehension, for example, for patients who understand fatigue as shortness of breath. | Sem cansaço (fraqueza)/Pior cansaço (fraqueza) possível |
| No nausea/Worst nausea imaginable | Sem náusea/Pior náusea possível | No nausea/Worst nausea possible | The word sickness, which is commonly used by patients in Brazil, has been added. This word was selected to add in parentheses after the term nausea. | Sem náusea (enjoo)/Pior náusea (enjoo) possível |
| No depression/Worst depression possible | Sem depressão/Pior depressão possível | No depression/Worst depression possible | The translated version was maintained (no changes). | Sem depressão/Pior depressão possível |
| No anxiety/Worst anxiety imaginable | Sem ansiedade/Pior ansiedade possível | No anxiety/Worst anxiety possible | The translated version was maintained (no changes). | Sem ansiedade/Pior ansiedade possível |
| No drowsiness/Worst drowsiness possible | Sem sonolência/Pior sonolência possível | No drowsiness/Worst drowsiness possible | The translated version was maintained (no changes). | Sem sonolência/Pior sonolência possível |
| Best appetite/Worst appetite possible | Melhor apetite/Pior apetite possível | Best appetite/Worst appetite possible | The translated version was maintained (no changes). | Melhor apetite/Pior apetite possível |
| Best feeling of well-being/Worst feeling of well-being | Melhor sensação de bem estar/Pior sensação de bem estar | Best sense of well-being/Worst sense of well-being | Considering the ESAS as an instrument with answers provided on a numerical visual scale, it is essential to clarify the notion of continuity. There was a fear that a patient with slight or moderate discomfort, for example, may respond with a 10 for "feeling of well-being" due to feeling sick. The panel considered it necessary to provide clarification on the opposite ends of the scale (best and worst possible). | Melhor sensação de bem estar/Pior sensação de mal estar possível |
| No shortness of breath/ Worst shortness of breath imaginable | Sem falta de ar/Pior falta de ar possível | No lack of air/ Worst lack of air possible | The translated version was maintained (no changes). | Sem falta de ar/Pior falta de ar possível |
| Best sleep/Worst sleep imaginable | Melhor sono/Pior sono possível | Best sleep/Worst sleep possible | The translated version was maintained (no changes). | Melhor sono/Pior sono possível |

^a”^Fadiga” (preferentially), and “cansaço” were both considered adequate terms.
