# Supplementary material for: The Brazilian Version of the Edmonton Symptom Assessment System (ESAS) Is a Feasible, Valid and Reliable Instrument for the Measurement of Symptoms in Advanced Cancer Patients
Source: PLoS One. 2015 Jul 8;10(7):e0132073. doi: 10.1371/journal.pone.0132073 (PMC4496067; doi:10.1371/journal.pone.0132073)
Supplement: S2 Table — (DOC) [file pone.0132073.s002.doc]

**­­**

**Escala de Avaliação de Sintomas de Edmonton (ESAS-Br)**

**Esta é uma escala de avaliação de sintomas. Você responderá a 10 itens com respostas que variam de 0 (mínima intensidade) a 10 (máxima intensidade). Por favor, circule o número que melhor descreve os seus sintomas nas últimas 24 horas:**

| **Sem**  **Dor** | **________________________________________________**  **0 1 2 3 4 5 6 7 8 9 10** | **Pior Dor**  **possível** |
| --- | --- | --- |
| **Sem cansaço (fraqueza)** | **________________________________________________**  **0 1 2 3 4 5 6 7 8 9 10** | **Pior cansaço (fraqueza) possível** |
| **Sem náusea (enjoo)** | **________________________________________________**  **0 1 2 3 4 5 6 7 8 9 10** | **Pior náusea (enjoo) possível** |
| **Sem depressão** | **________________________________________________**  **0 1 2 3 4 5 6 7 8 9 10** | **Pior depressãopossível** |
| **Sem ansiedade** | **________________________________________________**  **0 1 2 3 4 5 6 7 8 9 10** | **Pior ansiedade possível** |
| **Sem sonolência** | **________________________________________________**  **0 1 2 3 4 5 6 7 8 9 10** | **Pior sonolência possível** |
| **Melhor apetite** | **________________________________________________**  **0 1 2 3 4 5 6 7 8 9 10** | **Pior apetite possível** |
| **Melhor sensação de bem estar** | **________________________________________________**  **0 1 2 3 4 5 6 7 8 9 10** | **Pior sensação de mal estar possível** |
| **Sem falta de ar** | **________________________________________________**  **0 1 2 3 4 5 6 7 8 9 10** | **Pior falta de ar possível** |
| **Melhor sono** | **________________________________________________**  **0 1 2 3 4 5 6 7 8 9 10** | **Pior sono possível** |
